# Radiation Safety Practices and Occupational Exposure Among Brazilian Urologists: A National Survey

**DOI:** 10.1590/S1677-5538.IBJU.2026.0176

**Published:** 2026-04-25

**Authors:** Vicktor B. P. Pinto, Julyana K. M. Moromizato, Veridiana Andrioli, Rui W. Mascarenhas, Leonardo Seligra Lopes, Julia D. Souza, Rafael Ehrenfreund, Ricardo L. Vita, Bárbara V. L. A. Melão, Roni C. Fernandes, Cristiano M. Gomes, Karin M. J. Anzolch

**Affiliations:** 1 Faculdade de Medicina da Universidade de São Paulo Divisão de Urologia São Paulo SP Brasil Divisão de Urologia, Faculdade de Medicina da Universidade de São Paulo, São Paulo, SP, Brasil; 2 Universidade Estadual de Campinas Departamento de Cirurgia Divisão de Urologia Campinas SP Brasil Divisão de Urologia e Departamento de Cirurgia, Universidade Estadual de Campinas (UNICAMP), Campinas, SP, Brasil; 3 Universidade do Estado do Pará Divisão de Urologia Belém PA Brasil Divisão de Urologia, Universidade do Estado do Pará, Belém, PA, Brasil; 4 Centro Universitário FMABC Divisão de Urologia Santo André SP Brasil Divisão de Urologia, Centro Universitário FMABC, Santo André, SP, Brasil; 5 Hospital Militar da Região de São Paulo Clínica Urológica São Paulo SP Brasil Clínica Urológica, Hospital Militar da Região de São Paulo, São Paulo, SP, Brasil; 6 Hospital Moriah São Paulo SP Brasil Hospital Moriah, São Paulo, SP, Brasil; 7 Hospital Adventista de Manaus Departamento de Oncologia Manaus AM Brasil Departamento de Oncologia do Hospital Adventista de Manaus, Manaus, AM, Brasil; 8 Santa Casa de São Paulo Departamento de Urologia São Paulo SP Brasil Departamento de Urologia, Santa Casa de São Paulo, São Paulo, SP, Brasil; 9 Divisão de Urologia do Hospital Moinhos de Vento Porto Alegre RS Brasil Divisão de Urologia do Hospital Moinhos de Vento, Porto Alegre, RS, Brasil

## COMMENTS

Ionizing radiation is an integral component of contemporary urological practice (1). In daily routine, fluoroscopy is frequently used to support diagnostic procedures such as retrograde pyelography, voiding cystourethrography, and videourodynamics, as well as a wide range of therapeutic interventions (2). In addition to endourology and stone disease management, including ureteral stent placement, ureteroscopy, and percutaneous nephrolithotomy, fluoroscopic guidance may also be required in other settings, including sacral neuromodulation lead implantation and selected reconstructive or functional procedures (3).

Although indispensable for procedural guidance and decision-making, recurrent occupational exposure raises concerns about radiological safety in urology (1, 4). Occupational risk is primarily determined by cumulative radiation dose, exposure geometry, and the consistency of protective measures applied during fluoroscopy-guided procedures (2, 3). Interventional specialties are consistently among the most exposed groups in healthcare, and in urology the frequency of fluoroscopy-guided procedures across various clinical settings may translate into substantial cumulative exposure over years of practice (2, 4).

Core protection principles are grounded in the ALARA concept ("As Low As Reasonably Achievable") and aim to minimize radiation exposure by reducing fluoroscopy time, maximizing distance from the source, and optimizing shielding while maintaining adequate image quality (1, 5). In clinical practice, these principles translate into consistent use of personal protective equipment (PPE), including lead aprons, thyroid shields, gloves, and leaded eyewear, along with individual dosimetry and institutional processes to ensure equipment integrity and exposure monitoring (1, 2). However, real-world adherence to these measures remains heterogeneous and may be influenced by availability of protective equipment, workflow constraints, local safety culture, and the degree of institutional oversight (1, 6).

In Brazil, available data on occupational radiation exposure and radiation safety among urologists are still limited in scope, with previous nationwide efforts primarily focused on general behaviors and use of protective and monitoring equipment (6). Procedure-specific PPE use, availability of protective equipment in different practice settings, dosimeter adoption, and institutional monitoring processes have not been comprehensively evaluated, limiting the ability of institutions and professional societies to prioritize and implement targeted interventions.

Recent contributions from Brazilian urological research have also emphasized the importance of understanding real-world practice patterns and identifying gaps between recommendations and clinical practice across different domains of urology (7–9).

Therefore, we conducted a national, cross-sectional survey of Brazilian urologists to characterize self-reported sources and intensity of ionizing radiation exposure, PPE use across common fluoroscopy-guided procedures, availability of protective equipment, dosimeter use, institutional monitoring practices, perceived health consequences, and expectations regarding the role of the Brazilian Society of Urology (SBU) in promoting radiation safety. By defining current practices and gaps, this study aims to inform pragmatic, urology-centered strategies to strengthen radiation safety in Brazil and provide insights that may be applicable to similar practice settings worldwide.

## MATERIALS AND METHODS

### Study design and survey development

This study was conducted as a national, cross-sectional electronic survey designed to evaluate exposure to ionizing radiation, use of protective measures, and knowledge regarding radiation safety among Brazilian urologists. The survey was developed by urologists with expertise in different subspecialties, aiming to identify the most relevant aspects of radiation exposure and radioprotection in routine urological practice, as well as institutional support and safety policies.

The initial draft of the questionnaire was reviewed by a panel of experienced urologists representing functional urology and voiding dysfunction, benign prostatic hyperplasia, general urology, uro-oncology, endourology, andrology and sexual medicine, and pediatric urology. This multidisciplinary review aimed to ensure content relevance, clarity of wording, and clinical applicability of the proposed items. Prior to nationwide dissemination, the survey instrument underwent pilot testing among 12 practicing urologists to assess comprehensibility, estimated completion time, and technical functionality of the electronic platform. Minor revisions were made based on feedback obtained during this phase.

The questionnaire consisted predominantly of closed-ended, multiple-choice questions, with selected items allowing open-ended responses. The survey included sections addressing sociodemographic characteristics and professional practice patterns; sources and frequency of radiation exposure; use of PPE during residency and in current practice; availability of protective equipment in healthcare institutions; use of dosimeters and institutional control of radiation exposure; knowledge of radiation-related health risks and self-reported complications potentially related to radiation exposure; and perceptions regarding the role of the Brazilian Society of Urology in radiation safety education. The full questionnaire is available in Figure-1.

**Figure 1:**
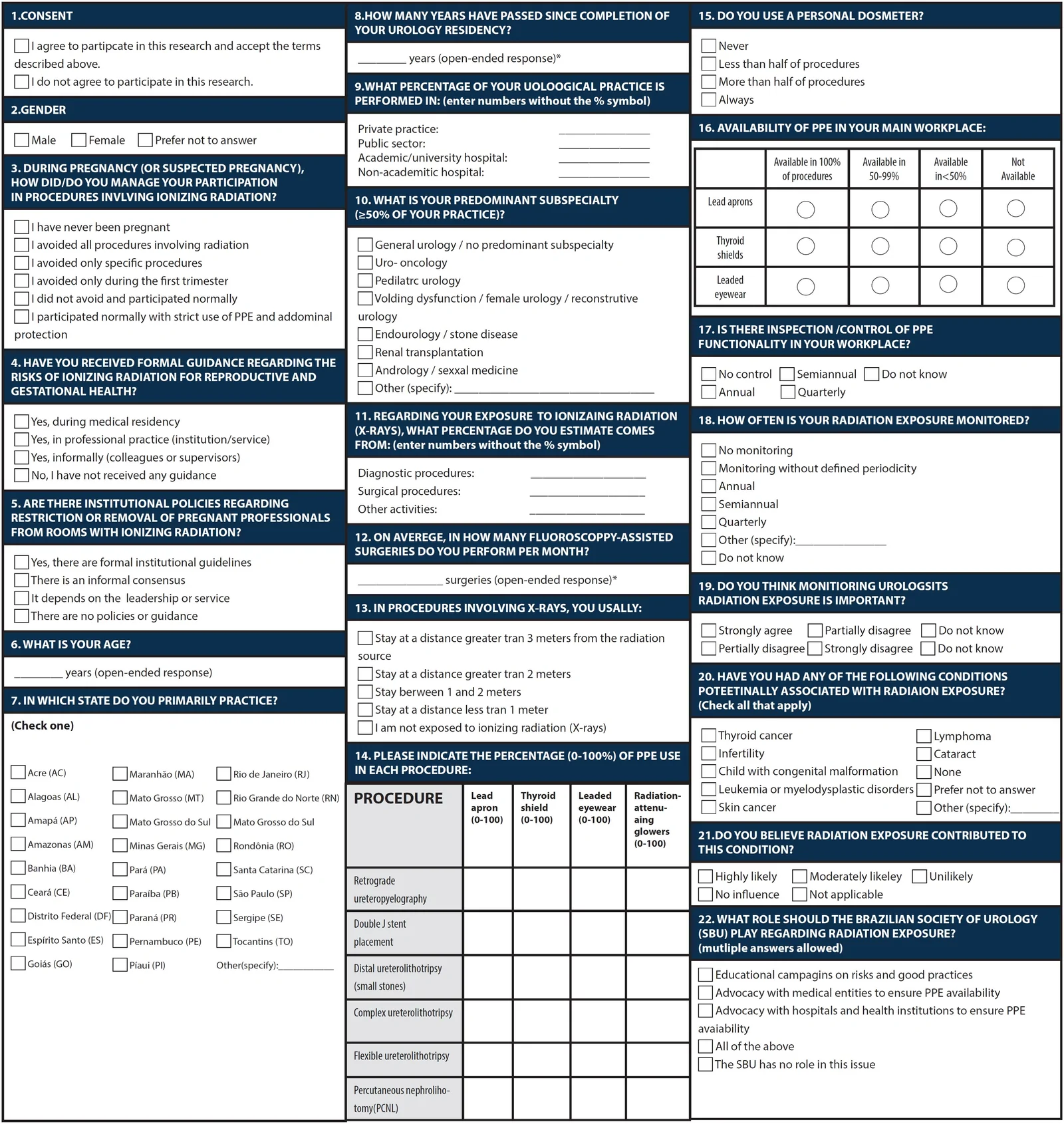
Survey questionnaire.

The questionnaire included items on demographics, professional practice, radiation exposure, use and availability of personal protective equipment, dosimetry, institutional monitoring, and perceptions regarding radiation safety. Open-ended responses were collected for selected items.

*Open-ended responses were collected for selected items*.

### Participants and survey distribution

All urologists registered with the Brazilian Society of Urology were eligible to participate in the study. Participation was voluntary and anonymous. The survey was distributed electronically through official SBU communication channels, including e-mail and instant messaging platforms.

The invitation was sent to approximately 3,000 eligible urologists registered with the Brazilian Society of Urology. The survey was open from July 9, 2025, to November 3, 2025. During this period, reminders were sent through SBU digital communication strategies to encourage participation. No financial or material incentives were offered for survey completion.

### Data collection and ethical considerations

Survey responses were collected using an online survey platform and stored in a secure digital database (SurveyMonkey®, Momentive Inc., San Mateo, CA, USA). No personally identifiable information was collected, ensuring participant anonymity. Before accessing the questionnaire, participants were required to read and electronically agree to an informed consent statement explaining the study objectives, voluntary nature of participation, data confidentiality, and minimal associated risks. The study was approved by the Institutional Review Board of our institution (project number CAAE: 84804624.3.0000.0068) and was conducted in accordance with national regulations and international ethical standards for research involving human participants.

### Variables and outcomes

Collected variables included age, sex, geographic region of practice, years since completion of residency, type of practice setting (private, public, or mixed), and predominant urological subspecialty. Radiation-related variables included self-reported sources of radiation exposure, frequency of fluoroscopy use, distance from the radiation source during procedures, use of PPE, availability of protective equipment in institutions, use of dosimeters, and existence of institutional monitoring of radiation exposure and equipment safety.

Knowledge-related variables assessed awareness of potential adverse health effects of ionizing radiation, while perception-related variables explored attitudes toward radiation safety and the perceived role of professional societies in promoting radioprotection.

### Statistical Analysis

Quantitative variables were described using measures of central tendency (means or medians) and dispersion (standard deviations or interquartile ranges), as appropriate. Qualitative variables were expressed as absolute numbers and proportions. Comparisons between groups were performed using Student's t test or analysis of variance (ANOVA) for continuous variables, and the chi-square test or Fisher's exact test for categorical variables.

Logistic regression analyses were conducted to explore factors associated with key outcomes related to radiation safety practices and protective behaviors. Odds ratios (ORs) with corresponding 95% confidence intervals (CIs) were calculated. Analyses were based on complete cases, with participants excluded from specific analyses when data for the variables of interest were missing.

All statistical tests were two-sided, and a p value < 0.05 was considered statistically significant. Statistical analyses were performed using GraphPad Prism, version 10.0.01 (GraphPad Software, San Diego, CA, USA).

## RESULTS

A total of 325 urologists (83.0% men) completed the survey, representing 10.8% of Brazilian urologists who received the survey link. The median age was 46 years [38–54]. Sociodemographic and practice characteristics are summarized in Table-1. Denominators vary across analyses due to missing responses.

**Table 1:** Participants' demographic and practice patterns.

Age (years)	46 [38–54]
**Gender**	
Male	269 (83.0%)
Female	55 (17.0%)
**Distribution by geographic Brazilian region**	
Southeast	162 (50.0%)
South	58 (17.9%)
Northeast	63 (19.4%)
Central-West	26 (8.0%)
North	15 (4.6%)
**Time since residency completion**	
< 10 years	108 (33.4%)
10–20 years	79 (24.5%)
> 20 years	136 (42.1%)
**Workplace (admitted more than one option)**	
Some affiliation with private practice	309 (95.1%)
Some involvement in the public sector	197 (60.6%)
University-affiliated hospitals	112 (34.5%)
Private hospitals	221 (68.0%)
Multidisciplinary clinics	94 (28.9%)
**Predominant urological subspecialty**	
General urology / no predominant subspecialty	114 (35.1%)
Endourology / stone disease	77 (23.7%)
Uro-oncology	63 (19.4%)
Voiding dysfunction / female urology	36 (11.1%)
Andrology / sexual medicine	18 (5.5%)
Pediatric urology	9 (2.8%)
Renal transplantation	5 (1.5%)
Other	3 (0.9%)

Values are presented as n (%) unless otherwise indicated. Age is reported as median [interquartile range]. Workplace variables allowed multiple responses; therefore, percentages may exceed 100%. Denominators may vary across variables due to missing responses.

Among respondents, 42.1% completed their urology residency more than 20 years ago, 24.5% completed it 10-20 years ago, and 33.4% completed it less than 10 years ago. Most respondents (95.1%) reported working at least part-time in private practice, while 197 (60.6%) reported some degree of involvement in the public sector. Additionally, 112 (34.5%) worked in university-affiliated hospitals, 221 (68.0%) in private hospitals, and 94 (28.9%) in multidisciplinary clinics.

The distribution according to predominant urological subspecialty is presented in Table-1, most participants reported general urology (35.1%), followed by endourology/stone disease (23.7%) and uro-oncology (19.4%), while 11.1% reported a predominant practice in voiding dysfunction/female urology.

### Exposure profile

In terms of self-reported exposure sources, most participants attributed the majority of their ionizing radiation exposure to surgical procedures (median 95% [80-100]), whereas a smaller proportion was attributed to diagnostic examinations (median 5% [0-10]) and other activities (median 0% [0-0]). Participants reported performing a median of 10 fluoroscopy-assisted surgeries per month [5-15]. Fluoroscopy workload differed significantly by predominant subspecialty (p<0.001), being highest among endourology/stone disease practitioners (median 15 [10-20.5]) compared with general urology (median 10 [6-15]), uro-oncology (median 6 [5-10]), and voiding dysfunction/female urology (median 6 [3-10]). During X-ray-guided examinations and procedures, most urologists reported standing at a distance <1 m (169/324, 52.2%) or 1-2 m (129/324, 39.8%) from the radiation source.

### Patterns of personal protective equipment (PPE)

The estimated use of PPE varied substantially according to the type of equipment and procedure (Table-2). Lead apron use was consistently high across all assessed procedures, with median values of 100% in all scenarios (IQR 90-100 or 100-100). Thyroid shield use was lower and more variable, with median use ranging from 90% [0-100] during double-J stent placement and distal ureterolithotripsy to 100% [50-100] in more complex endourological procedures and 100% [75-100] during percutaneous nephrolithotripsy. In contrast, leaded eyewear and radiation-attenuating gloves were rarely used in any of the evaluated procedures, with median use of 0% [0-0] throughout.

**Table 2:** Use of personal protective equipment (PPE) by procedure.

Procedure	Lead apron, median [IQR]	Thyroid shield, median [IQR]	Leaded eyewear, median [IQR]	Radiation-attenuating gloves, median [IQR]
**Retrograde ureteropyelography**	100 [100–100]	100 [0–100]	0 [0–0]	0 [0–0]
**Double-J stent placement**	100 [90–100]	90 [0–100]	0 [0–0]	0 [0–0]
**Distal ureterolithotripsy (small stones)**	100 [90–100]	90 [0–100]	0 [0–0]	0 [0–0]
**Complex ureterolithotripsy**	100 [100–100]	100 [50–100]	0 [0–0]	0 [0–0]
**Flexible ureterorenolithotripsy**	100 [100–100]	100 [50–100]	0 [0–0]	0 [0–0]
**Percutaneous nephrolithotripsy**	100 [100–100]	100 [75–100]	0 [0–0]	0 [0–0]

Values represent the self-reported percentage of PPE use (0–100%) during each procedure and are presented as median [interquartile range]. PPE: personal protective equipment.

Thyroid shield use varied according to time since residency completion, with lower adherence among respondents who completed residency >20 years ago (Kruskal-Wallis p=0.014; Spearman ρ=−0.175, p=0.0015). No significant association was observed for lead apron, leaded eyewear, or radiation-attenuating glove use (all p>0.20).

### Monitoring practices and institutional policies for radiation protection

Regarding individual dose monitoring, 57/324 (17.6%) reported using a dosimeter. Dosimeter use was more frequent among urologists reporting higher monthly volumes of fluoroscopy-assisted surgeries (≥10/month vs <10/month; OR 2.96, 95% CI 1.52-5.76; p=0.001).

All respondents who answered the question regarding the importance of monitoring agreed (fully or partially) that urologists’ radiation exposure should be controlled (302/302, 100%). However, the reported presence of institutional processes for PPE maintenance and verification was limited: 52/324 (16.0%) reported any periodic control (annual, semiannual, or quarterly), while 120/324 (37.0%) reported no control and 152/324 (46.9%) did not know. Similarly, regarding monitoring of individual radiation exposure, 202/324 (62.3%) reported that no monitoring is performed in their services, 73/324 (22.5%) did not know, and 49/324 (15.1%) reported some form of monitoring (with or without a defined periodicity).

Concerning PPE availability in the main workplace, lead aprons for surgeons were reported to be available in 100% of procedures by 232/322 (72.0%), and in 50-99% of procedures by 76/322 (23.6%). Thyroid shields were reported to be available in 100% of procedures by 165/321 (51.4%) and in 50-99% by 105/321 (32.7%). Consistent thyroid shield use was more frequent when thyroid shields were reported as available in 100% of procedures (OR 1.92, 95% CI 1.15-3.20; p=0.012). In contrast, leaded eyewear was reported as unavailable by 268/324 (82.7%).

### Pregnancy-related radiation safety practices

Among female respondents (n=55), 54 answered additional questions addressing reproductive and pregnancy-related radiation safety. Regarding participation in procedures involving ionizing radiation during pregnancy (or suspected pregnancy), most reported avoiding all radiation-exposing procedures (21/54, 38.9%), while 3/54 (5.6%) reported continuing to participate with strict PPE use and abdominal shielding, 3/54 (5.6%) reported avoiding exposure only during the first trimester, and 2/54 (3.7%) reported avoiding only specific procedures; 25/54 (46.3%) reported never having been pregnant. Only 6/54 (11.1%) reported having received formal counseling on the reproductive and gestational risks of ionizing radiation (during residency or in professional practice), whereas 27/54 (50.0%) reported only informal guidance and 21/54 (38.9%) reported no guidance. In current practice, formal institutional policies restricting or removing pregnant professionals from radiation rooms were reported by 9/53 (17.0%), while 11/53 (20.8%) described an informal consensus, 9/53 (17.0%) reported that this depended on leadership or service, and 24/53 (45.3%) reported no guidance or policy.

### Role of the Brazilian Society of Urology (SBU)

Most respondents supported an active role for the SBU in addressing ionizing radiation exposure. Suggested actions included educational campaigns on radiation risks and best practices (113/324, 34.9%), advocacy with medical entities (95/324, 29.3%), and advocacy with healthcare institutions to improve the availability of PPE (96/324, 29.6%). Notably, 276/324 respondents (85.2%) selected the option "all of the above," indicating support for a combined strategy encompassing education and institutional advocacy. Only 3/324 (0.9%) believed that the SBU should have no role in this matter. One participant did not respond to this question.

### Radiation-related health concerns

Regarding the development of clinical conditions potentially associated with ionizing radiation exposure, 32/324 (9.9%) reported at least one condition. The most frequently reported conditions were cataract (3.7%), thyroid malignancy (2.5%), infertility (2.2%), and skin cancer (2.2%), followed by having a child with congenital malformations (1.5%), leukemia and other myelodysplastic disorders (0.9%), and lymphoma (0.6%). Among respondents reporting any condition, 50.0% considered it highly or moderately likely that lifetime ionizing radiation exposure contributed to the development of their condition (Figure-2).

**Figure 2:**
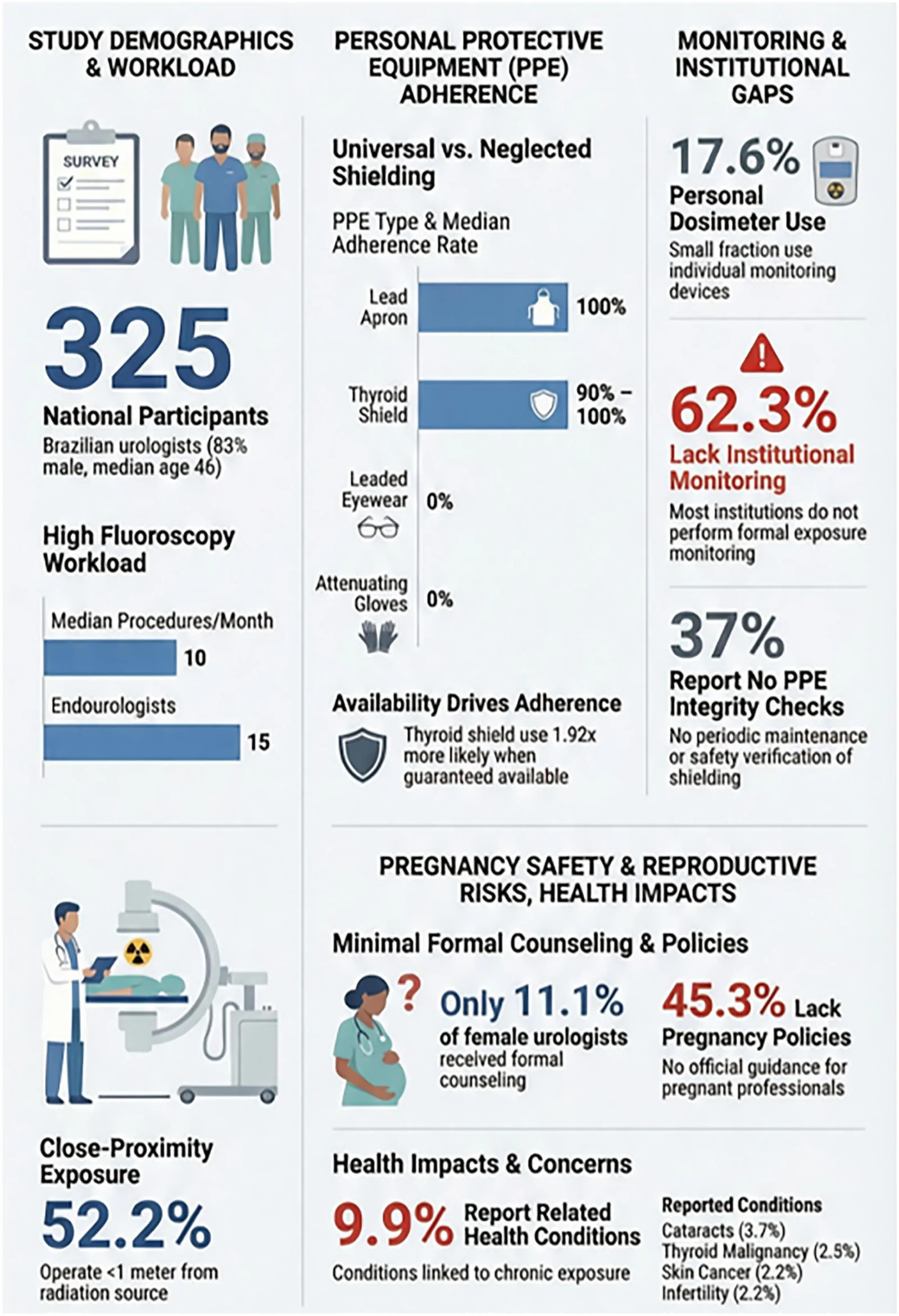
Patterns of Radiation Exposure, Personal Protective QSafety Gaps Among Brazilian Urologists.

PPE = personal protective equipment.

## DISCUSSION

This national survey provides a contemporary overview of radiation exposure patterns, protective behaviors, institutional monitoring practices, and pregnancy-related safety considerations among Brazilian urologists. The findings demonstrate a persistent gap between awareness of radiation risks and the consistent implementation of structured radioprotection strategies in daily practice. PPE use varied according to procedural complexity: lead apron adherence was nearly universal, and thyroid shield utilization was higher in complex endourological procedures. However, leaded eyewear and radiation-attenuating gloves were virtually absent. When compared with previous national data, partial improvement is evident: in the nationwide survey by Borges et al. (2015) (6), 84.4% of respondents reported consistent lead apron use and 53.9% reported thyroid protection. In the present study, conducted roughly a decade later, apron adherence approached universal levels and thyroid shield use demonstrated higher median rates. Nonetheless, the continued absence of eye protection and the low prevalence of dosimeter use indicate that radiation safety remains incomplete and that key anatomical risk sites (lens, hands, thyroid) are still insufficiently protected.

Individual dose monitoring represents one of the most critical deficiencies identified. Only 17.6% of respondents reported personal dosimeter use, more than 60% indicated the absence of institutional exposure monitoring, and nearly half were unaware of periodic PPE integrity verification. Although higher fluoroscopy workload was associated with greater dosimeter use, radiation protection cannot rely on subjective perception of exposure alone. As highlighted in recent educational reviews on radiation safety in urology (10), sustainable safety culture requires structured training, systematic monitoring, and institutional accountability. Contemporary dosimetry data reinforce this concern. Measurable scatter radiation exposure has been demonstrated across multiple professional roles during endourological procedures, with significant correlations between dose-area product and staff exposure (3). Systematic analyses have also shown substantial variability in effective radiation doses to urologists and have emphasized the importance of shielding and structured education in reducing occupational exposure by up to 94–100% (2). Together, these findings further suggest that cumulative exposure may be underestimated, particularly in high-volume settings.

The biological risks of chronic radiation exposure, including cataract formation, radiation-induced skin injury, and increased malignancy risk, are well documented (3, 4, 11, 12). Although causality cannot be established from self-reported data, the fact that 9.9% of respondents reported at least one medical condition potentially associated with radiation exposure underscores the perceived and potential occupational impact. Half of these respondents considered radiation exposure a likely contributing factor, highlighting not only biological concerns but also psychological and professional implications.

A notable and innovative aspect of this study is the detailed assessment of pregnancy-related radiation practices, an area that has been infrequently addressed in urological surveys. Formal counseling regarding reproductive risks was uncommon, and institutional policies restricting radiation exposure during pregnancy were inconsistently reported. Although most pregnant or previously pregnant respondents avoided radiation-exposing procedures, strategies varied and were frequently based on informal arrangements. This variability gains further relevance in light of the increasing representation of women in urology globally (13, 14). As the proportion of female urologists grows, the need for structured and transparent reproductive radiation safety frameworks becomes progressively more critical. Recent literature supports the feasibility of pregnancy-compatible fluoroscopic practice when protective strategies are rigorously applied. Koolik et al. (2025) emphasized adherence to ALARA principles with lead shielding and low-dose protocols (5), while Song et al. (2025) quantified uterine exposure during PCNL and demonstrated that active dose-reduction strategies allow procedures to remain within recommended fetal exposure thresholds (15). Together, these findings suggest that safe practice during pregnancy is achievable, but only within clearly defined institutional protocols that explicitly address role allocation, shielding, and dose monitoring.

Our data further indicate that radiation safety behavior is strongly influenced by infrastructure. The association between equipment availability and its use suggests that adherence is not solely an individual decision. The reported absence of leaded eyewear in most workplaces illustrates how structural limitations directly constrain compliance. Radiation protection therefore requires both professional awareness and institutional commitment, including investment in appropriate PPE and routine maintenance and monitoring processes.

The present study has several limitations. As a voluntary survey with a response rate of 10.8%, it is subject to selection and self-reporting bias, and respondents may not fully represent the broader Brazilian urologist population in terms of practice patterns, access to equipment, or attitudes toward radiation safety. However, this response rate is consistent with those commonly observed in large-scale web-based surveys among physicians, particularly when participation is voluntary and no incentives are provided. Importantly, the sample included urologists from all geographic regions of Brazil and across diverse practice settings, including public and private sectors as well as academic and non-academic institutions, supporting the heterogeneity of the sample and supporting the external validity of the findings. Objective radiation dose measurements were not performed and perceptions of exposure and risk may be distorted in either direction. We also did not perform stratified analyses according to geographic region, city size, or predominant practice setting (public vs private vs mixed), which may influence both access to protective equipment and institutional safety culture. No causal relationship between reported health conditions and radiation exposure can be established. Finally, although the survey provides a comprehensive national overview, these findings reflect the Brazilian context and cannot be directly extrapolated to other countries or healthcare systems without consideration of local infrastructure, regulations, and training environments.

Despite these limitations, this study has several strengths. It represents a comprehensive, contemporary national assessment of radiation exposure and radioprotection practices among Brazilian urologists, encompassing diverse practice settings and subspecialties through the institutional reach of the SBU. The survey instrument underwent multidisciplinary expert review and pilot testing, supporting content validity and clinical applicability. Importantly, the study evaluated not only personal protective equipment use but also institutional infrastructure, dosimetry practices, and pregnancy-related policies—domains that have seldom been examined simultaneously in prior surveys. By integrating behavioral and structural variables and identifying factors associated with protective practices, the findings provide data that may inform targeted educational and institutional strategies to enhance radiation safety in Brazilian urology.

Although foundational protective measures such as lead apron use are widely incorporated, comprehensive radiation safety culture remains underdeveloped. Systematic monitoring, broader PPE adoption, structured pregnancy policies, and institutional oversight are necessary to ensure that increasing reliance on fluoroscopy is accompanied by proportional commitment to occupational health and long-term risk mitigation.

## CONCLUSIONS

This national survey reveals that, although radiation protection is widely recognized as important, its structured application in Brazilian urology remains inconsistent. Limited use of dosimetry, heterogeneous institutional monitoring, inconsistent availability of PPE, and insufficiently defined pregnancy policies indicate that radioprotection is not yet fully integrated into routine practice. These findings reinforce the need for targeted educational initiatives and institutional standardization, supported by active engagement of professional societies and renewed emphasis on the ALARA principle to ensure sustained protection of both medical personnel and patients in fluoroscopy-guided urological care. Although focused on Brazil, these findings may be informative for other settings with similar resource constraints and practice patterns.

## Data Availability

All data generated or analysed during this study are included in the published article.

## ABBREVIATIONS

ALARA: As Low As Reasonably Achievable
CI: Confidence Interval
IQR: Interquartile Range
OR: Odds Ratio
PPE: Personal protective equipment
SBU: Brazilian Society of Urology (Sociedade Brasileira de Urologia)

## References

[1] Smith-Bindman R, Lipson J, Marcus R, Kim KP, Mahesh M, Gould R (2009). Radiation dose associated with common computed tomography examinations and the associated lifetime attributable risk of cancer. Arch Intern Med.

[2] De Coninck V, Hendrickx L, Mortiers X, Somani B, Emiliani E, Sener ET (2024). Radiation exposure of urologists during endourological procedures: a systematic review. World J Urol.

[3] Deininger S, Nairz O, Dieplinger AM, Deininger C, Lusuardi L, Ramesmayer C (2024). Real-time dosimetry in endourology: tracking staff radiation risks. Diagnostics (Basel).

[4] Hellawell GO, Mutch SJ, Thevendran G, Wells E, Morgan RJ (2005). Radiation exposure and the urologist: what are the risks?. J Urol.

[5] Koolik IM, Bock ME, Antonelli JA (2025). Managing radiation exposure to endourologists during pregnancy. Curr Urol Rep.

[6] Borges CF, Reggio E, Vicentini FC, Reis LO, Carnelli GR, Fregonesi A (2015). How are we protecting ourselves from radiation exposure? A nationwide survey. Int Urol Nephrol.

[7] Passarelli F, Basadonna LM, Ciamarra F, Bonacina E, Graps G, Sorba E (2026). Acute kidney injury following mini percutaneous nephrolithotomy for renal stones: predictors and follow-up evaluation in real-life setting. Int Braz J Urol.

[8] Mota PKV, Ferreira DB, Florencio RFD, Cohen DJ, Perrella R, Batagello CA (2024). Are very thin patients at a higher risk of complications when submitted to percutaneous nephrolithotomy?. Int Braz J Urol.

[9] Pellanda AB, Torricelli FCM, Denstedt J, Danilovic A, Marchini GS, Vicentini FC (2024). Endoscopic combined intrarenal surgery: best practices and future perspectives. Int Braz J Urol.

[10] Smith M, Thatcher MD, Davidovic F, Chan G (2024). Radiation safety education and practices in urology: a review. J Endourol.

[11] López PO, Dauer LT, Loose R, Martin CJ, Miller DL, Vañó E (2018). ICRP Publication 139: occupational radiological protection in interventional procedures. Ann ICRP.

[12] Patel R, Dubin J, Olweny EO, Elsamra SE, Weiss RE (2017). Use of fluoroscopy and potential long-term radiation effects on cataract formation. J Endourol.

[13] No authors (2023). Women in urology — closing the gap. Nat Rev Urol.

[14] Li A, Cheng Z, Hunt TC, Thavaseelan S, Cancian M, Bandari J (2025). Rising representation: trends in female urology residency and fellowship matches over the past decade. Am J Surg.

[15] Song S, Amasyali AS, Jhang D, Clark T, Cheng KW, Park K (2025). Radiating for two: quantifying radiation exposure to pregnant urologists during percutaneous nephrolithotomy. J Urol.

